# A puzzling homology: a brittle star using a putative cnidarian-type luciferase for bioluminescence

**DOI:** 10.1098/rsob.160300

**Published:** 2017-04-05

**Authors:** Jérôme Delroisse, Esther Ullrich-Lüter, Stefanie Blaue, Olga Ortega-Martinez, Igor Eeckhaut, Patrick Flammang, Jérôme Mallefet

**Affiliations:** 1 Research Institute for Biosciences, Biology of Marine Organisms and Biomimetics, University of Mons - UMONS, 23 Place du Parc, 7000 Mons, Belgium; 2 Museum für Naturkunde, Leibniz Institute for Evolution and Biodiversity Science, Invalidenstr. 43, 10115 Berlin, Germany; 3 Department of Marine Science, The Sven Lovén Centre for Marine Sciences – Kristineberg, University of Gothenburg, 45178 Fiskebäckskil, Sweden; 4 Marine Biology Laboratory, Université Catholique de Louvain, ELI, 3 Place Croix du Sud L7.04.06, 1348 Louvain-La-Neuve, Belgium

**Keywords:** bioluminescence, luciferase, evolution, echinoderm

## Abstract

Bioluminescence relies on the oxidation of a luciferin substrate catalysed by a luciferase enzyme. Luciferins and luciferases are generic terms used to describe a large variety of substrates and enzymes. Whereas luciferins can be shared by phylogenetically distant organisms which feed on organisms producing them, luciferases have been thought to be lineage-specific enzymes. Numerous light emission systems would then have co-emerged independently along the tree of life resulting in a plethora of non-homologous luciferases. Here, we identify for the first time a candidate luciferase of a luminous echinoderm, the ophiuroid *Amphiura filiformis*. Phylogenomic analyses identified the brittle star predicted luciferase as homologous to the luciferase of the sea pansy *Renilla* (Cnidaria), contradicting with the traditional viewpoint according to which luciferases would generally be of convergent origins. The similarity between the *Renilla* and *Amphiura* luciferases allowed us to detect the latter using anti-*Renilla* luciferase antibodies. Luciferase expression was specifically localized in the spines which were demonstrated to be the bioluminescent organs *in vivo*. However, enzymes homologous to the *Renilla* luciferase but unable to trigger light emission were also identified in non-luminous echinoderms and metazoans. Our findings strongly indicate that those enzymes, belonging to the haloalkane dehalogenase family, might then have been convergently co-opted into luciferases in cnidarians and echinoderms. In these two benthic suspension-feeding species, similar ecological pressures would constitute strong selective forces for the functional shift of these enzymes and the emergence of bioluminescence.

## 1. Introduction

Bioluminescence is a common phenomenon in marine ecosystems [1,2]. This ability to emit light seems to have evolved independently at least 40 times and at least 17 phyla have developed this ability [1–5]. Bioluminescence is used for (i) predation avoidance, (ii) luring prey, and (iii) intraspecific signalling during courtship and mating [2,5].

Chemically, bioluminescence is described as the oxidation of a substrate called luciferin by molecular oxygen [6,7]. The reaction, catalysed by an enzyme called luciferase, forms a molecular product in an electronically excited state, which is sufficiently energetic to result in the emission of a photon [2,8]. In some particular cases, luciferin, oxygen and the luciferase apoprotein are bound together in a ‘pre-charged’ compound [8,9], the so-called photoproteins. All known luciferases are oxygenases that use molecular oxygen to oxidize an organic substrate to generate energy-rich peroxidic intermediates. To date, several luminous systems have been described involving different combinations of enzymes, cofactors and luciferins [2,10]. Only a relatively small number of luciferins have been characterized from luminous marine organisms and some of them are shared by phylogenetically distant organisms [2,5,11,12]. This large phylogenetic coverage is partly explained by the transmission of the luciferin through the food chain. Coelenterazine, originally named for its presence in cnidarians [11,13], is the most widespread marine luciferin [2,5]. It occurs in radiolarians, cnidarians, ctenophores, chaetognaths, crustaceans, echinoderms and fish [8,12,14–18]. Coelenterazine is naturally found in conjunction with both photoproteins and luciferases *stricto sensu*. Conversely to the relatively restricted diversity of luciferins, many different luciferases have already been identified and these enzymes are supposed to be taxon-specific [2,19].

In echinoderms, luminous species have been described in four of the five classes (Crinoidea, Holothuroidea, Asteroidea and Ophiuroidea) but no light emitting species were found in the class Echinoidea [20,21]. In ophiuroids, out of the 2200 described species [22,23], about 220 were tested for light emission and more than 75 were proved to be luminous [21]. The emission of light in brittle stars seems to be mainly or exclusively linked to an anti-predation function [20,24–28]. Despite the relatively common occurrence of luminous echinoderms, only two ophiuroid species, *Amphiura filiformis* and *Ophiopsila californica*, have been investigated biochemically. The former luminesces with a coelenterazine–luciferase system whereas the latter emits light with a photoprotein system [9,29]. Up to now, no luciferase *sensu lato* sequence has been obtained for echinoderms and those enzymes remain enigmatic in this phylum.

**Figure 1.**
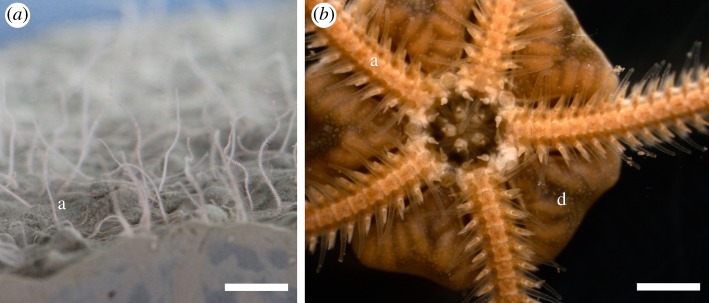
The brittle star *Amphiura filiformis.* (*a*) Arms of *A. filiformis* emerging out of the sediment (in aquarium; scale bar, 1 cm). (*b*) Oral view of *A. filiformis* (scale bar, 2 mm).

The present work aimed at the identification of the luciferase of *A. filiformis*. Sequences homologous to known luciferases were searched in the genome and an adult transcriptome of *A. filiformis*. This ophiuroid, a dominant species on most sublittoral soft bottoms in Europe, is characterized by an infaunal lifestyle with the individuals feeding on suspended particles by extending two arms in the water column (figure 1). Genome and transcriptome analyses made it possible to highlight putative luciferase genes and their expression in the arms of *A. filiformis.* In particular, sequences similar to the luciferase of the luminous sea pansy *Renilla* (RLuc) attracted our attention. The corresponding RLuc-like protein was specifically detected by immunofluorescence in the arm spines of the brittle star, which were identified as the photogenic areas by videography with brilliance intensification and macrophotography. RLuc-like sequences were also identified in genomic and transcriptomic databases from non-luminous echinoderms and metazoans. However, only extracts from the arms of *A. filiformis* produced light in the presence of coelenterazine, raising interesting questions about the other putative functions of these luciferase-like enzymes.

## 2. Results and discussion

### 2.1. *Renilla* luciferase-like proteins are expressed in both luminous and non-luminous organisms

Thanks to new genomic and transcriptomic data, the number of available sequences for echinoderms, including the brittle star model species *A. filiformis,* is increasing exponentially [30–34]. In order to highlight the potential luciferase of this luminous ophiuroid species, sequences similar to all known light emitting luciferases were searched in a reference transcriptome of adult arm tissue [35]. We found transcripts similar to four luciferase queries: the *Renilla* luciferase (RLuc) (Cnidaria, Anthozoa), the *Suberites* luciferase (Porifera), the firefly luciferases (sequences from multiple species, Arthropoda, Insecta) and the *Watasenia* candidate luciferases (Mollusca, Cephalopoda). Summarized BLAST results are presented in the electronic supplementary material table S1. Among these different sequences, we focused our attention on three mRNA sequences with a strong homology with the luciferase gene of the Anthozoa *Renilla* sp. (RLuc). Indeed, the luminous system of *A. filiformis* is considered as physiologically similar to the one of *Renilla reniformis*, with the use of an identical luciferin, coelenterazine, and a comparable coelenterazine-specific luciferase activity [8,36,37] making this RLuc-like enzyme a good luciferase candidate for *A. filiformis*. Additionally, *Suberites* and firefly luciferases and *Watasenia* candidate luciferases all correspond to the ‘insect-type luciferases’ which, with the exception of *Watasenia* candidate luciferase, are not coelenterazine-specific [11,38,39]. Moreover, these luciferases are known to be homologous to the ubiquitous acyl-CoA ligases that are involved in metabolism of all organisms from bacteria to metazoans [39–42]. It is not surprising therefore to find sequences similar to acyl-CoA ligases in *A. filiformis*.

**Figure 2.**
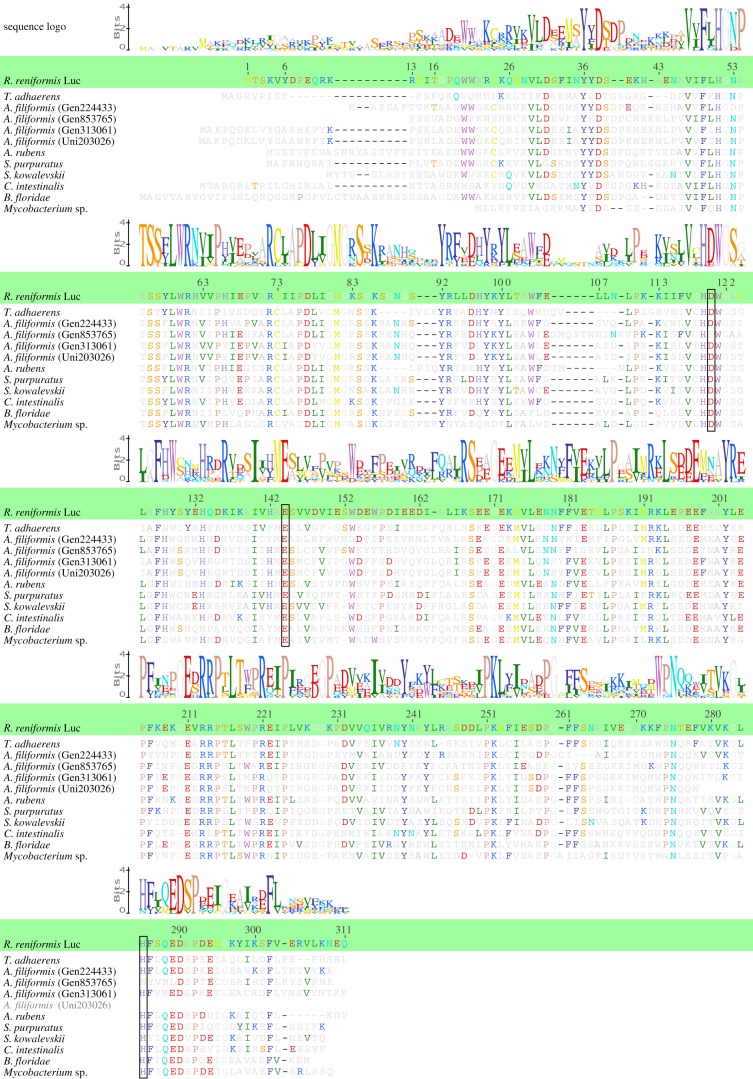
Multiple sequence alignment of the *Renilla* luciferase (RLuc), deuterostomian luciferase-like proteins (echinoderms: *Amphiura filiformis, Asterias rubens, Strongylocentrotus purpuratus*; hemichordate: *Saccoglossus kowalevskii*; urochordate: *Ciona intestinalis*), placozoan luciferase-like protein (*Trichoplax adhaerens*) and bacterial haloalkane dehalogenase proteins (*Mycobacterium* sp.). Alignment was performed using Geneious software (MAFFT alignment). Conserved amino acids relative to RLuc are coloured in all sequences. The amino acid triad known as the active site in haloalkane dehalogenases and RLuc is framed in black. This amino acid triad is conserved in all luciferase-like sequences we identified except in *A. filiformis* Gen853765. Sequence accession numbers: *T. adhaerens*, XP002116677; *R. reniformis*, AAA29804.1; *S. purpuratus*, DspA XP794172.2; *A. rubens*, Ar_comp22488; *B. floridae*, XP002611539.1; *C. intestinalis*, XP002127127.1; *S. kowalevskii*, XP002738321.1, XPE002730984.1; *Mycobacterium* sp., WP067006024.

RLuc is known to catalyse the oxidation of coelenterazine to yield coelenteramide, carbon dioxide and blue light [14]. It shows a characteristic alpha/beta-hydrolase fold [43] and was found to have a high level of similarity in tertiary structure and to be homologous to the bacterial haloalkane dehalogenases, which are primarily hydrolase enzymes cleaving a carbon–halogen bond in halogenated compounds [44–48]. This degree of similarity is somewhat surprising considering that haloalkane dehalogenases are hydrolases and RLuc is an oxygenase. A bacterial haloalkane dehalogenase (outgroup, *Mycobacterium* sp.) is presented in the alignment of figure 2. *Mycobacterium* haloalkane dehalogenase shares high sequence identity (38%) and similarity (55%) with the sequence of RLuc (electronic supplementary material, table S2). Asp120, Glu144 and His285 together form the active site of RLuc and homologous bacterial haloalkane dehalogenases, and mutations within this site deactivate the enzymes [44,49,50]. The amino acid triad is conserved in most luciferase-like sequences we identified (see legend of figure 2 for additional information). Cys 73 is also required for the activity of RLuc [44,51], and this specific amino acid was found in all metazoan luciferase-like sequences while it is absent in numerous microbial haloalkane dehalogenases (figure 2 and electronic supplementary material, figure S1).

In addition to the three identified arm transcripts, genomic searches highlighted 13 RLuc-like sequences in the draft genome of *A. filiformis*. All RLuc-like sequences and the reciprocal BLASTx results are presented in the electronic supplementary material, files S1 and table S3, respectively. RLuc-like predicted protein sequences share up to 44% amino acid identity and a general similarity of up to 62% when compared with RLuc (electronic supplementary material, table S2). BLAST searches also permitted identification of multiple sequences homologous to RLuc in the sea urchin genome and in several echinoderm transcriptomes belonging to all five classes [32,35,52] (electronic supplementary material, file S2). Predicted sea urchin luciferase-like sequences have up to 48% of identity and up to 66% of similarity to RLuc with a coverage of 96% (electronic supplementary material, table S2). RLuc-like genes were also detected in other deuterostome genomes including one urochordate (*Ciona intestinalis*), one cephalochordate (*Branchiostoma floridae*) and one hemichordate (*Saccoglossus kowalevskii*). Although these three species are not luminous, these sequences share more than 44% of identity with the RLuc sequence (electronic supplementary material, table S2). On the contrary, RLuc-like genes are rare in ‘non-deuterostomia’ databases. Besides RLuc, no similar sequence was found in cnidarians but homologous sequences were detected in *Trichoplax adhaerens* (Placozoa) and *Capitella teleta* (Annelida). RLuc-like genes were also reported in another annelid species (*Hermodice carunculata*) [53]. The alignment of representative RLuc-like sequences found in online databases is presented in the electronic supplementary material, figure S1.

**Figure 3.**
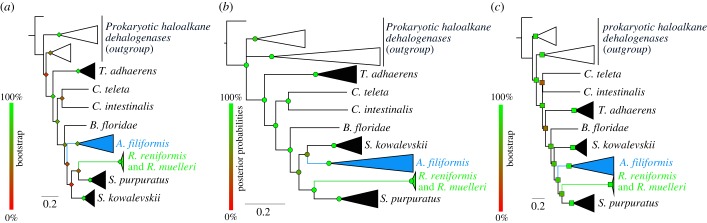
Phylogenetic reconstructions of RLuc-like sequence relationships based on maximum likelihood (*a*), Bayesian (*b*) and distance (*c*) methods. Bacterial haloalkane dehalogenases were taken as outgroup. Bootstrap (for *a,c*) and posterior probability (for *b*) values are colour-coded from red (0%) to green (100%). Short/partial sequences were not used for the analyses. The analyses included sequences from *Amphiura filiformis* (Gen224433, Uni203026, Gen313061 and Gen853765), *Renilla reniformis* (AAA29804), *Renilla muelleri* (AAG540941), *Strongylocentrotus purpuratus* (XP788943, XP794172, XP794218, XP798042 and XP792159), *Saccoglossus kowalevskii* (XP002730984 and XP002738321), *Ciona intestinalis* (XP002127127), *Branchiostoma floridae* (XP002611539), *Capitella teleta* (ELT94308), *Trichoplax adhaerens* (XP002116677 and XP002116678) and the haloalkane dehalogenases from *Roseobacter* (EBA17164.1, and WP009810405), *Phaeobacter* (WP054461824), *Thalassobius mediterraneus* (WP058317896.1), *Sulfitobacter pontiacus* (WP064216788), *Shimia* (WP054002384), *Mycobacterium* sp. (WP066929894), *Geobacter daltonii* (WP012647128), *Dehalococcoidia bacterium* (KPJ48875), *Myxococcales bacterium* (KPK14642) and *Algiphilus aromaticivorans* (WP043766976).

Phylogenetic relationships between RLuc, haloalkane dehalogenases and metazoan RLuc-like sequences were estimated using maximum-likelihood, Bayesian and distance methods (figure 3). The distinction between bacterial haloalkane dehalogenases and the ‘RLuc-like proteins containing group’ is clear. Within this second group, the topology of the trees appears variable and uncertain. In all cases, RLucs cluster with echinoderm sequences. Interestingly, *T. adhaerens* sequences do not cluster with RLuc sequences but instead branch basally to the cluster containing other metazoan RLuc-like sequences.

### 2.2. Only RLuc-like proteins from luminous organisms present a luciferase activity

**Figure 4.**
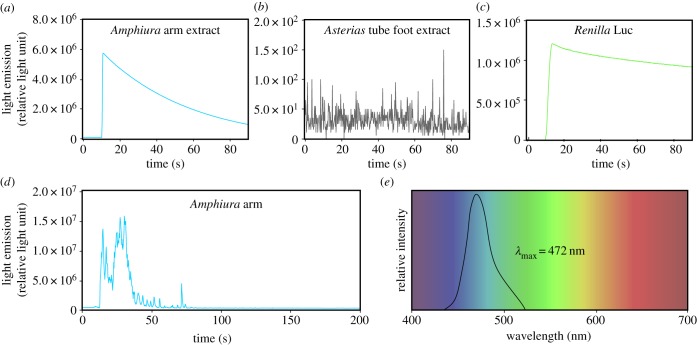
*In vitro* luciferase activity of echinoderm extracts (*a–c*) and *in vivo* light emission in *A. filiformis* (*d,e*). Luminometry tests of luciferase activity performed on *A. filiformis* arm extracts (*a*), on *A. rubens* tube foot extracts (*b*), and on purified RLuc (*c*), after coelenterazine addition. (*d*) Light emission of an arm in an adult *A. filiformis* measured by luminometry (RLU: relative light unit). (*e*) Bioluminescence spectral emission of *A. filiformis* measured by microspectrophotometry.

As we identified RLuc-like proteins in both luminous and non-luminous echinoderms, one can wonder whether all these proteins have luciferase activity and, therefore, whether the light emission capacity of the organisms could be in fact limited by the presence/absence of the luciferin substrate. Dietary acquisition of luciferins has indeed been proved experimentally or suggested for numerous species [2,16,54–57]. In *A. filiformis,* bioluminescence decreases with time in captivity (J Delroisse 2015, personal observations) suggesting that coelenterazine or a precursor might be acquired through feeding in this species. Non-luminous species could thus simply not have access to coelenterazine. To measure luciferase activity, a specific assay was performed on crude protein extracts from the arms of *A. filiformis*, on the one hand, and from the tube feet of the sea star *Asterias rubens*, on the other hand, to test their ability to trigger light emission in the presence of the coelenterazine substrate (figure 4*a–c*)*.* Purified RLuc was used as a positive control (figure 4*c*). *Asterias rubens* was chosen because we identified RLuc-like sequences in that species at the transcriptomic level (see above). Additionally, we immunodetected the RLuc-like protein in *A. rubens* tube feet using a polyclonal antibody specifically directed against RLuc (electronic supplementary material, figure S2), confirming the presence of the protein in tube feet*.* After coelenterazine addition to the protein extracts, light emission was detected in the *A. filiformis* arm extracts (figure 4*a*) while no bioluminescence was observed in the *A. rubens* tube foot extracts (figure 4*b*), supporting the idea that the sea star RLuc-like protein does not exhibit a light triggering activity. Interestingly, Fortova *et al.* [58] also reported the absence of light emission after coelenterazine addition, indicating the absence of a luciferase function, for a particular RLuc-like protein from the sea urchin *Strongylocentrotus purpuratus*. This protein, DspA, was recently characterized as the first haloalkane dehalogenase of non-microbial origin [58]. It seems therefore that RLuc-like proteins would possess only the haloalkane dehalogenase function in sea urchins. Similarly, we could hypothesize that the haloalkane dehalogenase function, defined as the ancestral function, might be also conserved in other non-luminous species.

In parallel to this *in vitro* assay, *A. filiformis* bioluminescence was characterized *in vivo*, at the organism level, by triggering light emission in anaesthetized individuals, using entire individuals, arm fragments or discs. The light emission kinetics was measured by luminometry using the technique of Mallefet *et al.* [59]. When chemically stimulated, the brittle star *A. filiformis* emitted flashes of light for about 30 s (figure 4*d*). The measurement of bioluminescence spectral emission by microspectrophotometry showed a maximum emission peak (*λ*_max_) at about 472 nm (figure 4*e*).

### 2.3. Light production and RLuc-like protein expression are co-localized at the level of the spines in *Amphiura filiformis*

**Figure 5.**
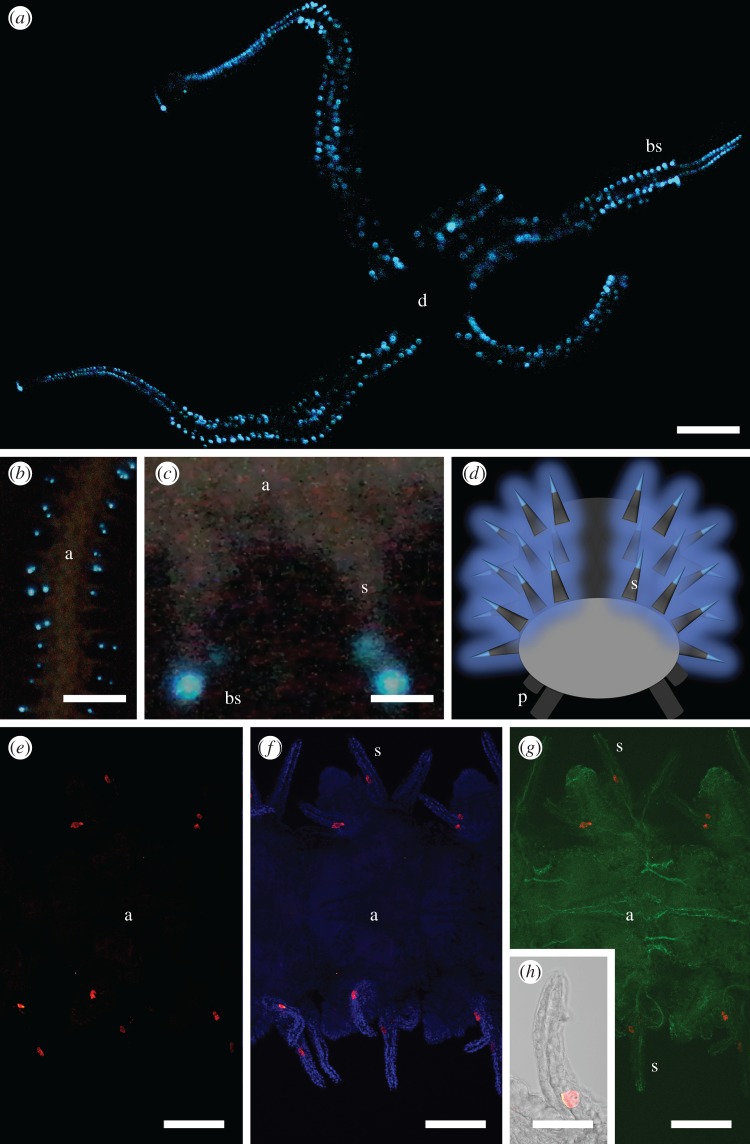
Light emission in *A. filiformis* and immunodetection of RLuc-like proteins. (*a*) Aboral view of an individual during KCl stimulation (scale bar, 0.5 cm). (*b*) Detail of the arm light emission (scale bar, 1 mm). (*c*) Detail of the spine emission (scale bar, 150 µm). (*d*) Schematic view of the arm bioluminescence. (*e–h*) Immunolocalization of RLuc-like proteins (red) and acetylated alpha tubulin (green) in an arm portion of *A. filiformis*. Nuclear DAPI staining is in blue (scale bar, 200 µm). (*h*) Immunolocalization of RLuc-like proteins in a single spine (scale bar, 40 µm; a, arm; d, disc; s, spine; bs, luminous blue spot; p, podia.

Detection of the luminous areas of *A. filiformis* by macrophotography and videography with brilliance intensification demonstrated blue emission on the arms and the absence of emission on the disc (figure 5*a*), as previously reported in the literature [60,61]. It is noteworthy that, under normal conditions, arms are the only part of the animal body present in the water column, the disc being buried in the sediment. From observations of flashing individuals or arms, it could be seen that light originated from areas located at the spine tips only (figure 5*b–d*). All spines emitted light when the arm was chemically stimulated. Conversely, a mechanical stimulation triggered only a local light emission around the stimulated area.

Immunodetections were performed on whole mount arm tissues of *A. filiformis* using polyclonal antibodies specifically directed against RLuc. Immunoreactive cells, presumably corresponding to photocytes, were detected only in the stroma, i.e. the inner part of the spines, where they form cell clusters (figure 5*e–g*). This organization is congruent with the description in some historical studies of putative photocytes present at the base of the spines of *A. filiformis*, their cell body tapering into an elongated cell process running all along the spine [60]. The specific expression of RLuc-like proteins in the spines together with our *in vivo* observations of light emission at the level of these organs strongly support that these proteins are the candidate luciferases of *A. filiformis*. Additional data on the biochemical characterization of the enzyme will be needed to confirm the ability of the candidate luciferase of *A. filiformis* to emit light *in vitro*. Although the fine morphology of the luminous areas and the photocyte organization still remain unknown in this species, our findings open the way for further studies on its bioluminescence. In particular, as photocytes have been identified, their ultrastructure could be investigated to describe potential microsources involved in the light emission, as previously performed for other luminous species [62–64].

### 2.4. New insights on luciferase evolution

**Figure 6.**
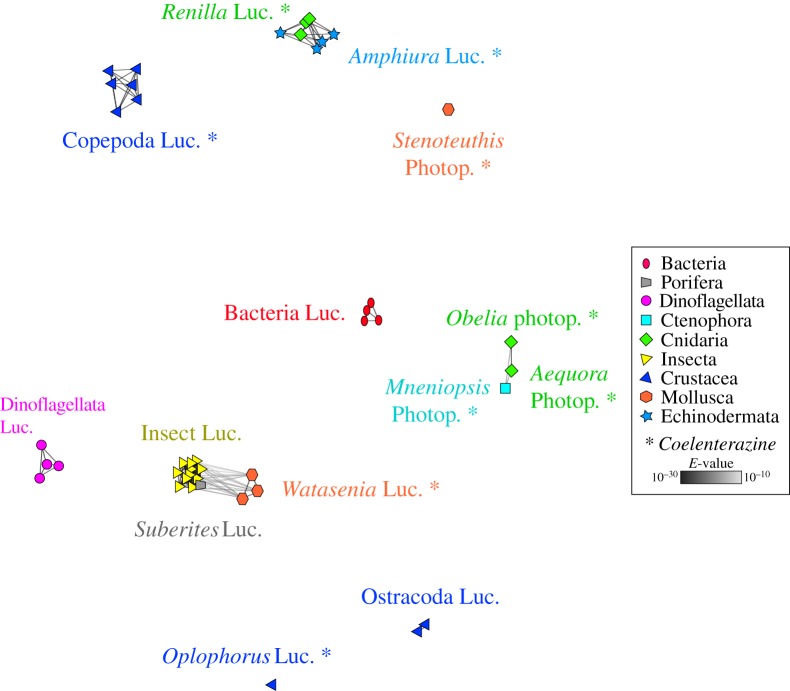
BLAST cluster map of all known luciferase/photoproteins. Node coloration is based on taxonomy. Lines correspond to BLAST connections of *p*-value < 1 10^−3^.

Bioluminescence relies on the oxidation of a luciferin substrate catalysed by a luciferase enzyme, luciferins and luciferases being generic terms covering a large variety of substrates and enzymes [6,7]. Whereas luciferins can be shared by phylogenetically distant organisms which feed on organisms producing them (as exemplified by the widespread occurrence of coelenterazine in the marine world [2,11,16]; figure 6), luciferases have been thought to be lineage-specific enzymes [2,19]. Numerous light emission systems would then have co-emerged independently along the tree of life resulting in a plethora of non-homologous luciferases. Global sequence-similarity-based clustering analyses conducted using all known light-emitting proteins including luciferases and photoproteins available in online databases and the RLuc-like proteins from *A. filiformis* partially confirm this hypothesis. Indeed, in the resulting cluster map, homologous luciferases are usually grouped in taxonomically homogeneous clusters which appear unrelated one with another (figure 6). As far as coelenterazine-specific luciferases are concerned, several have been described [45,65–70] but no large sequence similarity has been seen among them so far (figure 6). This suggests that many enzymes evolved to use coelenterazine for light emission [2,5]. However, paradoxically, our study shows the presence of two similar luciferases in two phylogenetically distant species (*Renilla* sp. and *A. filiformis*; figure 6). Recently, similar cases were described for the sponge *Suberites domuncula* and for the squid *Watasenia scintillans*, in which firefly-type luciferases were demonstrated to be involved in light emission [71,72] (figure 6). Luciferases could be co-opted from other genes with other functions and share high similarity with these primary genes, as was shown in the case of insect bioluminescence in which luciferases evolved from acyl-CoA ligases [39].

**Figure 7.**
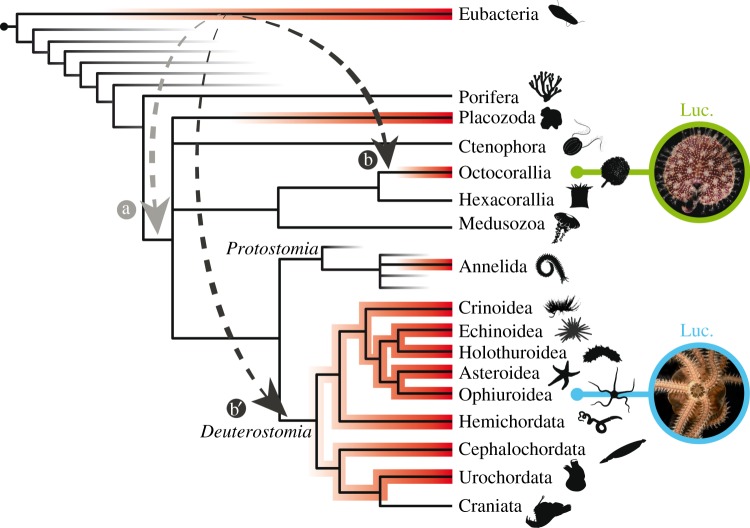
Distribution of haloalkane dehalogenases/RLuc-like proteins in metazoans and associated evolutionary hypotheses. The presence of haloalkane dehalogenases/RLuc-like proteins in specific taxa is shown in red (based on BLAST analyses performed on nr/nt/TSA databases). Arrows illustrated hypothetical gene transfers. Tree based on the Tree of Life Project (http://tolweb.org). Organism silhouettes were extracted from the PhyloPic open database (phylopic.org). *Renilla reniformis* picture courtesy of Dr Alvaro Esteves Migotto (University of Sao Paulo).

In the case of *Renilla/Amphiura*, luciferases presumably evolved from haloalkane dehalogenases that have a hydrolase function. Some authors speculated that a horizontal gene transfer could be responsible for the presence of that bacterial-type homologous protein in Octocorallia [44], which would explain the high similarity between them and why we were not able to find any related sequence in other cnidarians. Horizontal gene transfers are known to play an important role in eukaryotic evolution leading to the acquisition of novel traits [73]. Our results highlight the possibility that the horizontal gene transfer occurred in the common ancestor of cnidarians and echinoderms (*hypothesis a*). Additionally, multiple independent horizontal gene transfers from prokaryotes to multiple marine metazoans could also explain the sparse presence of haloalkane dehalogenases/RLuc-like proteins in metazoans (*hypothesis b*). Horizontal gene transfers from prokaryotes to metazoans, with secondary gene transfers from metazoans or other metazoans, could also be hypothesized (*hypothesis c*). However, our phylogenetic analyses support a monophyly of metazoan haloalkane dehalogenases/RLuc-like proteins making the *hypothesis a* more probable. *Hypotheses a* and *b* are illustrated in figure 7. This contradicts classical theories suggesting that bioluminescence arose from oxygenases involved in the removal of oxygen (by-product of oxygen detoxification when photosynthetic oxygen started to rise in the atmosphere) or involved in oxidation of increasing levels of unsaturated and aromatic compounds during early life history [74,75]. As we showed here and as it was already shown for insect bioluminescence [39], all luciferases did not originate from oxygenases but, rather, selective pressure drove the emergence of new oxygenase functions. A coelenterazine-specific luciferase could have evolved from an enzyme that catalyses a non-related reaction, as coelenterazine chemiluminesces easily in aprotic solvents [44]. The enzyme would only have to provide a hydrophobic environment for coelenterazine to achieve some low level of bioluminescence [44].

We hypothesize that the haloalkane-dehalogenase function constituted the metazoan ancestral state, which shifted to luciferase in octocorals (lineage of *Renilla* sp. and potentially luminous sea-pens in general) and brittle stars (lineage of *A. filiformis;* Echinoderm, Ophiuroidea). Haloalkane dehalogenases were therefore co-opted in luciferases in these two specific lineages. In *A. filiformis*, the apparent recent duplications of luciferase-like genes could suggest the co-occurrence of both luciferase and haloalkane dehalogenase functions. Alternatively, luciferase/haloalkane dehalogenase enzymes could be bifunctional and potentially have a context-dependent bioactivity as observed for some insect luciferases [42]. This last hypothesis is, however, not corroborated by our luminometry assay results or by a previous study performed on sea urchins [58]. *Renilla* sp. and *A. filiformis* thus possess similar and homologous luciferases to catalyse the photogenous reaction. A co-emergence happened between these two luminous systems using the same compounds under similar environmental pressure. The ecological similarities between the sea pansies (*R. muelleri, R. reniformis*) and the brittle star *A. filiformis*, such as the benthic position on soft sediments and the suspension-feeding strategy, would presumably allow dietary acquisition of coelenterazine from planktonic organisms. The predation pressure would positively select the emergence of the bioluminescence function endowing these slow moving organisms with an efficient anti-predation strategy.

## 3. Material and methods

### 3.1. Organisms sampling

Specimens of *A. filiformis* (Müller 1776) were collected in the vicinity of the Lovén Center (Kristineberg, Sweden) at 25–40 m in depth by using a mechanical grab. The brittle stars were carefully rinsed out of the sediment, and intact specimens were kept in sediment and running deep-seawater (12–14%, 32‰ salinity). The individuals were transported to the Marine Biology Laboratory of the University of Louvain-La-Neuve, and kept alive in a closed-circuit marine aquarium filled with a mixture of natural and artificial recirculating seawater until required. Individuals of *A. rubens* Linnaeus, 1758 were collected on rocks during low tide at Audresselles (Opal Coast, France) and Roscoff (Brittany, France). They were transported to the Biology of Marine Organisms and Biomimetics laboratory of the University of Mons, where they were kept in a marine aquarium with closed circulation (13°C, 32‰ salinity).

### 3.2. *In silico* analyses

To identify the putative luciferase of *A. filiformis,* reference luciferase/photoprotein sequences from various luminous organisms obtained from open-access NCBI online databases (http://www.ncbi.nlm.nih.gov) were used in a ‘tBLASTn/reciprocal BLASTx’ approach. These sequences are listed in the electronic supplementary material, file S1. Homologous sequences to reference luciferases were first searched using tBLASTn in an adult arm transcriptome of *A. filiformis* [35]. Arms are known to be the light emitting body parts in *A. filiformis.* Candidate matches were then used as queries in a reciprocal BLASTx search against online databases in order to highlight sequences with high similarity with potential luciferases. Secondarily, luciferase-like sequences of interest found in the *A. filiformis* transcriptome were also searched in the draft genome of *A. filiformis* (http://www.echinobase.org/Echinobase/AfiBase) using local tFASTx searches (v. 36.3.4) on the *A. filiformis* dataset (957 749 165 residues and 1 407 676 sequences). Exon–intron predictions were performed on potential luciferase sequences found in the *A. filiformis* draft genome using the GENSCAN Web Server (http://genes.mit.edu/GENSCAN.html). A similar approach was used to look for luciferase-like sequences in multiple echinoderm databases such as the genome of *S. purpuratus* (Echinoidea) and a tube foot transcriptome of *A. rubens* (Asteroidea) [32,35,52,76]*.*

*In silico* translations were performed on all luciferase-like sequences found in transcriptomic databases using the Expasy translate tool (http://web.expasy.org/translate/). A multiple amino acid alignment of RLuc-like protein sequences was performed using MAFFT algorithm (v. 7.017, E-INS-i, Scoring matrix: Blosum 62, Gap open penalty: 1,53) and refined using MUSCLE algorithm in Geneious software (v. 8.1.5., www.geneious.com). For phylogenetic and similarity matrix reconstructions, alignment trimming was performed using BMGE software (http://mobyle.pasteur.fr/cgi-bin/portal.py). Pairwise sequence identity and similarity were calculated from multiple sequence alignments using SIAS web tool (http://imed.med.ucm.es/Tools/sias.html).

Based on the trimmed alignment, phylogenetic analyses were conducted using maximum-likelihood, Bayesian and distance methods. Maximum-likelihood phylogenetic tree was reconstructed using the PhyML software (v. 3.1/3.0 aLRT) [77]. Best-fit model analysis was previously conducted using Smart Model Selection (http://www.atgc-montpellier.fr/sms). The WAG substitution model was selected assuming an estimated proportion of invariant sites (of 0.041) and four gamma-distributed rate categories to account for rate heterogeneity across sites. The gamma shape parameter was estimated directly from the data (gamma = 1.078). Reliability for internal branch was assessed using the bootstrapping method (100 bootstrap replicates). The Bayesian phylogenetic tree was reconstructed using MrBayes software (v. 3.2.3) [78] using the GTR + G model. Four Markov Chain Monte Carlo (MCMC) chains were run for 3 000 000 generations reaching a split frequency value inferior to 0.01. The first 25% sampled trees were discarded as ‘burn-in’. Finally, a 50% majority rule consensus tree was constructed. In parallel, neighbour joining tree was reconstructed using BioNJ. The JTT model was used for substitution while the default gamma shape parameter was set to 1. Reliability of the tree was assessed using the bootstrapping method (1000 bootstrap replicates). All known luciferase/photoprotein sequences and new potential *A. filiformis* luciferase sequences were also analysed using a sequence-similarity-based clustering approach based on BLASTp e-values and using the CLANS software [79].

### 3.3. Luminometry assays and spectral measurements

In order to measure luciferase activity, an *in vitro* luminometry assay was performed in which echinoderm fresh tissue extracts were subjected to coelenterazine addition following the procedure classically used in luciferase characterization [8]. Arms from the brittle star *A. filiformis* and tube feet from the sea star *A. rubens* were homogenized in a fivefold (w : v) volume of distilled water, in order to extract the potential luciferase-like enzymes as described in Shimomura [8]. The extracts were kept on ice and in the dark between experiments and all reactions were performed within 1 h after extraction. Crude extracts were tested for their capability to react with purified coelenterazine (NanoLight, working dilution of 0.044 g l^−1^ in methanol) by recording light emission. The assay was done as follows: 20 µl of the tissue extract was added to 180 µl of Tris–HCl buffer (Tris–HCl 0.01 mol l^−1^, NaCl 0.5 mol l^−1^, pH 7.4). In another tube, 5 µl of coelenterazine was added to 195 µl of the same buffer. The background emission of the catalyst tube was recorded for 10 s, then the luciferin mixture was quickly injected and the recording was continued for 3 min. Measurements were performed using a FB12 luminometer (Berthold Detection System) coupled with the FB12 Sirius software v. 1.5. The potential weak self-emission of both reagents was independently controlled. As a positive control, coelenterazine was tested for its capability to react with purified *Renilla* luciferase (NanoLight, working dilution of 0.2 g l^−1^ in Tris–HCl buffer; Tris–HCl 0.01 mol l^−1^, NaCl 0.5 mol l^−1^, pH 7.4).

An *in vivo* assay was also conducted to measure the kinetics of bioluminescence in *A. filiformis*. Animals were first anaesthetized by immersion for 3 min in 3.5% w/v MgCl_2_ in artificial seawater (ASW: 400.4 mmol l^−1^ NaCl, 9.9 mmol l^−1^, CaCl_2_, 9.6 mmol l^−1^, KCl 52.3 mmol l^−1^, MgCl_2_ 27.7 mmol l^−1^, Na_2_SO_4_ 20 mmol l^−1^ Tris, pH 8.3). Arms were isolated from the disc and rinsed in filtered seawater. Light emission of entire individuals or dissected body parts was triggered using a v/v mixture of seawater and KCl 400 mmol l^−1^ solution according to the method of Mallefet *et al.* [59]. Light responses were recorded using the FB12 Berthold Luminometer linked to a laptop computer.

In order to localize the bioluminescent areas, fresh and anaesthetized organisms were used for luminous areas detection by videography with brilliance intensification and high sensitivity macrophotography. Emission spectra were measured with an optical fibre coupled to a minispectrophotometer (Hamamatsu Photonics K.K. TM-VIS/NIR: C10083CA, Hamamatsu-City, Japan; precision: 6 nm) positioned perpendicularly to the photogenic tissue at a distance of 1 mm.

### 3.4. Luciferase immunodetection

Based on the high similarity between luciferase-like sequences found in the genomic and transcriptomic databases and the *Renilla* luciferase, we performed immunodetections on arms of *A. filiformis* using a commercial anti-RLuc polyclonal antibody (Genetex GTX125851, generated against the full-length RLuc protein). Arms were anaesthetized using 7% MgCl_2_ in an equivolumic mixture of filtered seawater and distilled water. Arm pieces were fixed in 4% paraformaldehyde in phosphate-buffered saline (PBS: 0.05 M PB/0.3 M NaCl, pH 7.4) for 30–60 min at room temperature and decalcified in an equivolumic mixture of ascorbic acid 2% and NaCl 0.3 M. They were then rinsed in PBS and blocked in the same buffer containing 0.25% bovine serum albumin, 0.1% Triton X-100 and 0.05% NaN_3_ for 30 min at room temperature. Anti-RLuc antibodies were diluted in PBS (with a final dilution of 1 : 250). After an overnight incubation at 4°C, tissues were rinsed in PBS and then incubated in a 1 : 500 dilution of Alexa Fluor conjugated goat anti-rabbit IgG (Molecular Probes) for 2 h at room temperature. After several washes in PBS, specimens were mounted in an antifading medium containing a glycerin/PBS mixture and examined using a Zeiss 510 metaconfocal microscope. Projections shown in the present study were produced by recording confocal image stacks and projecting them in the *z*-axis using Fiji. The specificity of the immunofluorescent labelling was confirmed by control experiments using exactly the same procedure but omitting either the primary or the secondary antibodies. Double labelling was also performed using a second round of incubation of primary antibody, namely anti-acetylated alpha-tubulin (SIGMA).

## Supplementary Material

Table S1: BLAST searches of known luciferase-like in the arm transcriptome of A. filiformis

## Supplementary Material

Table S2: Similarity/identity matrix of RLuc-like predicted proteins

## Supplementary Material

Table S3: Reciprocal BLAST results of A. filiformis RLuc-like sequences in NR database (Genbank)

## Supplementary Material

File S1: A. filiformis RLuc-like sequences (cDNA/DNA) (fasta format)

## Supplementary Material

File S2: All Luc Like (fasta format)

## Supplementary Material

Figure S1: Multiple protein alignment of RLuc-like predicted proteins

## Data accessibility

All sequence data are listed in the electronic supplementary material, S1 and S2.
