# Supplementary figures and images for: A puzzling homology: a brittle star using a putative cnidarian-type luciferase for bioluminescence
Source: Open Biol. 2017 Apr 5;7(4):160300. doi: 10.1098/rsob.160300 (PMC5413902; doi:10.1098/rsob.160300)

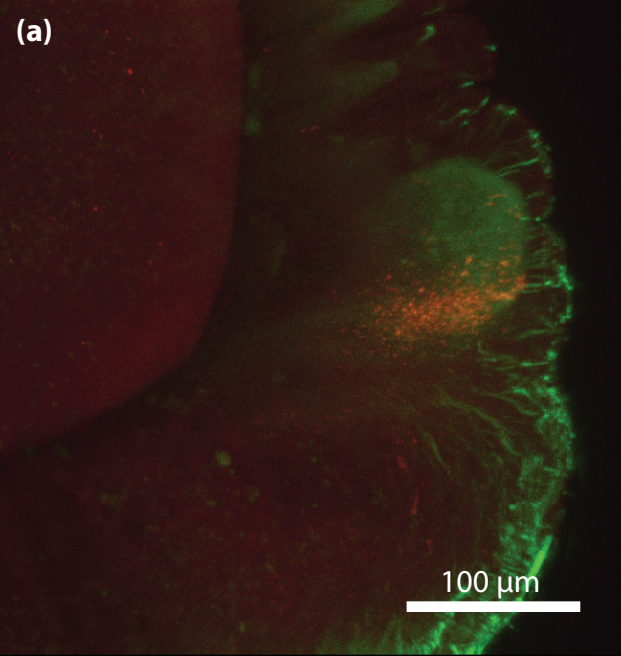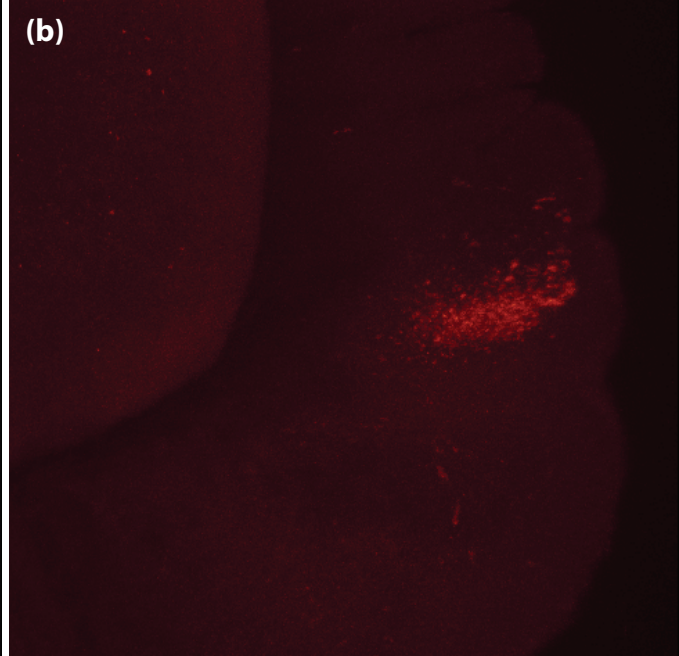

Supplement: Figure S1: Multiple protein alignment of RLuc-like predicted proteins [file rsob160300supp6.pdf]
